# The beneficial role of vitamin D in obesity: possible genetic and cell signaling mechanisms

**DOI:** 10.1186/1475-2891-12-89

**Published:** 2013-06-25

**Authors:** Khanh vinh quốc Lương, Lan Thi Hoàng Nguyễn

**Affiliations:** 1 Vietnamese American Medical Research Foundation, 14971 Brookhurst Street, Westminster, CA 92683, USA

## Abstract

The prevalence rates of overweight and obesity are considered an important public issue in the United States, and both of these conditions are increasing among both children and adults. There is evidence of aberrations in the vitamin D-endocrine system in obese subjects. Vitamin D deficiency is highly prevalent in patients with obesity, and many studies have demonstrated the significant effect of calcitriol on adipocytes. Genetic studies have provided an opportunity to determine which proteins link vitamin D to obesity pathology, including the vitamin D receptor, toll-like receptors, the renin-angiotensin system, apolipoprotein E, vascular endothelial growth factor, and poly (ADP-ribose) polymerase-1. Vitamin D also exerts its effect on obesity through cell-signaling mechanisms, including matrix metalloproteinases, mitogen-activated protein kinase pathways, the reduced form of nicotinamide adenine dinucleotide phosphate, prostaglandins, reactive oxygen species, and nitric oxide synthase.

In conclusion, vitamin D may have a role in obesity. The best form of vitamin D for use in the obese individuals is calcitriol because it is the active form of the vitamin D_3_ metabolite, its receptors are present in adipocytes, and modulates inflammatory cytokine expression.

## Background

Obesity is an emerging health problem of growing importance. There is evidence of aberrations in the vitamin D-endocrine system in obese subjects [1], such as increases in serum parathyroid hormone (PTH), urinary cyclic adenosine 3,5′-monophosphate (cAMP), renal tubular reabsorption of calcium, and circulating 1α, 25-hydroxyvitamin D_3_ (1,25OHD_3_) and a decrease in serum 25-hydroxyvitamin D_3_ (25OHD) levels. In young adults, the dietary plus supplemental vitamin D intake was inversely related to the development of incident metabolic syndrome over 20 years of follow-up [2]. Vitamin D deficiency is common in children in West Virginia and is associated with increasing age and obesity [3]. Vitamin D deficiency is an independent risk factor for obesity and abdominal obesity in women [4]. Obese women transfer less 25OHD to offspring than normal-weight women, despite similar serum levels; maternal obesity and vitamin D sufficiency are associated with cord-blood vitamin D insufficiency [5]. Visceral adipose tissue is negatively associated with plasma 25OHD concentrations in South Asians [6]. Total body fat has been shown to be a negative predictor of 25OHD levels in women even after controlling for age, lifestyle, and PTH in Germany [7]. Vitamin D deficiency has been associated with obesity, visceral obesity, hypertriglyceridemia, and metabolic syndrome in Korean children [8]. Body mass index (BMI) is inversely associated with the increase in the serum 25OHD levels in response to vitamin D supplementation [9]. The expression of vitamin D-metabolizing enzymes has been demonstrated in human adipose tissue. Plasma 25OHD increased by 27% after weight loss in the obese individuals. The expression levels of the 25-hydroxylase CYP2J2 and the 1α-hydroxylase CYP27B1 were decreased by 71% and 49%, respectively, in the subcutaneous adipose tissue of obese subjects [10], suggesting that adipose tissue, which can be dynamically altered during obesity and weight loss, has the capacity to metabolize vitamin D locally. Furthermore, calcitriol directly regulates adipocyte 11β-hydroxysteroid dehydrogenase type 1 (11β-HSD-1), generating active cortisol from inactive cortisone, with expression and cortisol production in human adipocytes *in vitro*[11], suggesting a potential role for calcitriol in visceral adiposity. In a 12-week double-blind randomized clinical trial, cholecalciferol supplementation resulted in a statistically significant decrease in body fat mass in healthy and obese women compared with the placebo group [12]. In addition, vitamin D_3_ supplementation also improved insulin sensitivity in apparently healthy, middle-aged, centrally obese men [13]. In our previous publication, we discussed the role of vitamin D in obesity [14]. These findings suggest that vitamin D may play a role in obesity. Herein, we further discuss the potential role of vitamin D in obesity, along with possible genetic and cell signaling mechanisms.

### Genetic factors that relate to vitamin D and obesity

Genetic studies provide an opportunity to link molecular variations with epidemiological data. DNA sequence variations, such as polymorphisms, exert both modest and subtle biological effects. Vitamin D exhibits immune-modulatory and anti-proliferative effects through the vitamin D receptor (VDR) in disease states. The VDR is present in adipose tissue and may contribute to the action of vitamin D and its analogs in adipocytes. Specific calcitriol-binding is evident in pre-adipocyte 3T3-L1 cells, but there is no evidence of specific calcitriol-binding in mature adipocytes [15]. This finding is consistent with the idea that any influence of vitamin D on adipogenesis would likely be exerted early in the pre-adipocyte to adipocyte transition when more VDR was available. However, Blumberg *et al*. [16] have further defined the molecular mechanism by which unliganded VDR and calcitriol-liganded VDR regulate adipogenesis. They also demonstrated that VDR is expressed early in adipogenesis. VDR were expressed sequentially at high, but short-lived, levels beginning at 30 min and lasting for only a few hours after adipogenic activation [17,18]. A functional vitamin D response element was identified in the murine Insig-2 promoter, and its potential role is involved in the differentiation of 3T3-L1 preadipocytes [19]. VDR-knockout mice have also displayed atrophy of adipose tissue surrounding the prostate and mammary glands [20,21]. Under normocalcemic conditions, VDR-null mice displayed less body fat mass and lower plasma triglyceride and cholesterol levels compared with wild-type (WT) mice. When placed on a high-fat diet, VDR-null mice displayed a slower growth rate and accumulated less fat mass globally than WT mice, even when their food intake and intestinal lipid transport capacity were the same as WT mice [22-24]. In one study, transgenic mice with targeted expression of human VDR in the adipose tissue developed obesity due to reduced energy expenditure [25]. Taken together, these data confirm an important role of the VDR in the control of adipocyte metabolism and the regulation of energy metabolism. Variations at the VDR locus have been associated with susceptibility and progression to several diseases. *VDR* gene polymorphisms have been linked to higher susceptibility to vitamin D deficiency in children and adolescents [26]. The *VDR Taq*I allele is associated with obesity [27,28]; *Bsm*I and *Apa*I *VDR* genes are also significantly associated with overweight and obesity [29], and the *Bsm*I *VDR* polymorphism appeared to influence body mass index [30]. These observations suggested that alterations of *VDR* function may play a role in patients with obesity.

Toll-like receptors (TLRs) are a group of glycoproteins that function as surface trans-membrane receptors. These receptors are involved in the innate immune response to exogenous pathogenic microorganisms. Substantial evidence exists supporting the important role of TLRs in the pathogenesis of obesity. Both TLR-2 and TLR-4 expression are increased in adipose tissue in patients with obesity and type 2 diabetes mellitus [31]. A high-fat diet- or leptin deficiency-induced obesity up-regulated the expression of TLR1-9 and TLR11-13 in murine adipose tissue. The extent of the obesity-induced up-regulation of most *TLR* genes and related pro-inflammatory signaling cascades was much greater in the epididymal adipose tissues than in the subcutaneous fat tissues of the mice with diet-induced obesity [32]. These results suggest that TLRs and the related pro-inflammatory signaling molecules that are overexpressed in enlarged adipose tissues may play an important role in the obesity-associated phenomenon of meta-inflammation. In addition, adipose stores may play a dynamic role in the regulation of inflammation and innate immunity in human subjects via modulation of the TLR/Nuclear Factor-kappaB (NF-ĸB) regulatory pathway [33]. TLR-4-deficient 10ScN mice were selectively protected against obesity-induced by diets high in saturated fat. Moreover, macrophage infiltration and monocyte chemotactic protein-1 (MCP-1) transcript abundance were lower in the adipose tissue of 10ScN mice fed an HFP diet [34]. C3H/HeJ mice, which have a loss-of-function mutation in *TLR*-*4*, were protected against the development of diet-induced obesity [35] and were less susceptible to fat-induced inflammation and insulin resistance [36]. The inhibition of TLR-2 expression improved insulin sensitivity and signaling in the muscle and white adipose tissue of mice fed a high-fat diet [37]. These findings suggest that TLRs are a key modulator of the crosstalk between the inflammatory and metabolic pathways. Furthermore, there is an association between *TLR*-*2* and *TLR*-*4* polymorphisms and serum levels of tumor necrosis factor-alpha and its soluble receptors in obese children [38], suggesting that serum cytokine levels may be affected by *TLR* polymorphisms in obese children. In contrast, vitamin D deficiency increases the expression of the hepatic mRNA levels of TLR-2, TLR-4, and TLR-9 in obese rats [39]. However, calcitriol suppresses the expression of TLR-2 and TLR-4 mRNA and protein in human monocytes and triggers hypo-responsiveness to pathogen-associated molecular patterns [40]. Calcitriol has also been shown to down-regulate intracellular TLR-2, TLR-4 and TLR-9 expression in human monocytes [41]. Interestingly, TLR activation up-regulates the expression of VDR and 1α-vitamin D hydroxylase in human monocytes [42]. Taken together, these data indicate that vitamin D may have a role in obesity via modulating the TLR pathways.

The primary function of the renin-angiotensin system (RAS) is to maintain fluid homeostasis and to regulate blood pressure. Angiotensin-converting enzyme (ACE) is a key enzyme in the RAS and converts angiotensin (AT) I to the potent vasoconstrictor AT II [43]. A local RAS has been described in adipose tissue. Human adipose tissue expresses angiotensinogen and RAS enzymes [44]. Angiotensinogen, ACE and type 1 AT receptor genes are widely expressed, both in human adipose tissue and in cultured human adipocytes [45]. Renin is synthesized and secreted by culture 3T3-L1 cells in a regulated manner [46]. The renin receptor protein is specifically synthesized in the stromal portion of human adipose tissue in both isolated inter-adipocyte stromal cells and in stromal areas [47]. Moderate weight loss reduces plasma renin and aldosterone levels [48], suggesting an effect of weight loss on the juxta-glomerular secretion of renin. The RAS has been implicated in a variety of adipose tissue function. AT II increases lipogenesis in cultured human and murine cells [49], and angiotensinogen-knockout mice display decreased weight gain and decreased adipocyte cell size [50]. The deletion of the AT_2_ receptor reduces adipose cell size and protects from diet-induced obesity and insulin resistance [51]. AT_2_ receptor deficiency attenuates adipocyte differentiation and decreases adipocyte number in atherosclerotic mice [52]. These results suggest that AT_2_ receptor stimulation in adipose tissue is involved in the improvement of adipocyte differentiation and adipose tissue dysfunction in atherosclerotic models. Local renin angiotensin expression regulates human mesenchymal stem cell differentiation to adipocytes. Combined treatment with exogenous AT II and the AT II type 1 receptor blocker valsartan further inhibited adipogenesis [53]. Obese Zucker rats exhibited a marked up-regulation of AT_1_ receptor mRNA expression [54]. ACE inhibition and AT_1_ receptor blockade prevented fatty liver and fibrosis in obese Zucker rats [55]. These findings suggest that the RAS plays a role in obesity. The RAS activity may be modified by variants of the genes coding functional proteins of this pathway. Among hypertensive subjects with metabolic syndrome, the presence of *ACE* polymorphisms, including *TT235*, *MM174*, *DD*, and *CC1166* genotypes, could be a risk factor for central obesity and dyslipidemia [56]. In response to low-energy diets in obese women, the reduction in body fat was significantly lower in patients with the *ACE D*/*D* genotype than the *I*/*I* plus *I*/*D* genotype [57]. There is an association of *ACE* gene polymorphisms with BMI in the hypertensive Tunisian population [58]. In the Olivetti Prospective Heart Study, the *ACE I*/*D* polymorphism was a significant predictor of overweight and abdominal adiposity in men, while *DD* homozygosity was associated with larger increases in body weight and blood pressure in aging persons, and with higher overweight incidence [59]. There was a significant association of the *I*/*D* polymorphism with obesity, but only in Caucasian men [60]. It is possible that hormonal differences between sexes may alter the influence that the *ACE I*/*D* polymorphism has on weight gain. However, there is also an interaction between vitamin D and the RAS. Lower 25OHD levels and higher BMI values have been associated with higher plasma renin and aldosterone concentrations in Indian subjects with hypertension [61]. Vitamin D metabolites have been inversely associated with circulating renin [62]. Genetic disruption of the *VDR* results in overstimulation of the RAS with increasing renin and angiotensin II productions, leading to high blood pressure and cardiac hypertrophy. Treatment with captopril reduced cardiac hypertrophy in VDR-knockout mice [63]. The inactivation of 1α-hydroxylase, inhibiting calcitriol production, also leads to the development of hypertension, cardiac hypertrophy and impaired cardiac function, along with an up-regulation of the RAS in both renal and cardiac tissues, which could be reduced with calcitriol treatment [64]. In contrast, VDR over-expression suppresses renin expression in the juxtaglomerular cells of mice independently of PTH and calcium [65]. These findings suggest that calcitriol may function as an endocrine suppressor of renin biosynthesis. Calcitriol suppresses renin gene transcription by blocking the activity of the cyclic AMP response element in the renin core promoter [66] and decreases ACE activity in bovine endothelial cells [67]. There was a positive association identified between 25OHD and the vascular sensitivity to AT II in obese Caucasians with hypertension [68]. Vitamin D_3_ therapy in obese subjects with hypertension modified renal plasma flow, mean arterial pressure, and tissue sensitivity to AT II similar to ACE inhibition [69]. Taken together, these findings indicate that the RAS is activated in obese patients and that vitamin D may play a role in patients with obesity by modulating the RAS.

Apolipoprotein E (apoE) is a plasma protein and a key regulator of cholesterol and lipid metabolism. ApoE mRNA and protein can be found in human and rodent adipose tissue [70,71]. The infusion of AT II into mice for 3 days significantly reduced apoE expression in adipocytes from freshly isolated adipose tissue. In isolated human adipocytes, treatment with AT II significantly reduced cellular and secreted apoE (by 20-60%). However, a specific AT_1_ receptor blocker, valsartan, eliminated the effect of AT II on adipocyte apoE expression [72]. In *in vivo* and *in vitro* experiments, endogenous adipocyte apoE expression modulated adipocyte lipid metabolism and adipocyte gene expression [71]. Endogenous adipocyte apoE expression has important implications for the acquisition of substrate from triglyceride-rich lipoprotein particles such as very-low-density-lipoprotein (VLDL) [73]. High expression of apoE impairs lipid storage and promotes cell proliferation in human adipocytes [74]. ApoE-knockout adipocytes are lipid-poor and triglyceride synthesis is lower compared to wild-type adipocytes in the absence and presence of extracellular VLDL. Macrophage-derived apoE ameliorates dyslipidemia and atherosclerosis in lean and obese apoE-deficient mice [75,76], suggesting that apoE plays a role in regulating dyslipidemia. In comparison with *ApoE3* mice, *ApoE2* mice had elevated fasting plasma lipid and insulin levels and displayed prolonged postprandial hyperlipidemia accompanied by increased granulocyte number and inflammation 2 hours after being fed a lipid-rich meal. In comparison with *ApoE3* mice, the *ApoE2* mice also presented increased adiposity when maintained on a Western-type, high-fat, high-cholesterol diet [77]. ApoE2-expressing mice were hyperlipidemic and had increased gonadal fat pad and adipocyte sizes compared with apoE3 mice. In isolated cells, however, the expression of the apoE2 isoform produced defective lipogenesis and increased triglyceride hydrolysis [78]. The *ApoE4* allele is associated with higher serum vitamin D levels [79]. The *ApoE4* allele is associated with a low bone mass in postmenopausal Japanese women [80]. The common *ApoE* polymorphism has a complex effect on bone metabolism in peri-menopausal Danish women during five years follow-up. Namely, among women not receiving hormone replacement therapy (HRT), those with *ApoE2* have a lower rate of bone mineral loss in the femoral neck and hip regions than other women, whilst among women receiving HRT, those with *ApoE4* gained more bone mineral than other women [81]. Calcitriol has been known to induce macrophages to exhibit specific saturable receptors for LDL and acetyl-LDL. The LDL receptor of calcitriol-induced macrophages has been found to exhibit specificity for apoB- and E-containing lipoproteins [82]. In *apoE*-knockout mice, an animal model with dyslipidemia, high oxidative stress, and pronounced atherosclerosis after uninephrectomy, the animals developed reduced plaque growth and calcification with vitamin D analog treatment (paricalcitol) compared with control groups [83,84]. Although vitamin D deficiency is associated with an unfavorable lipid profile in cross-sectional analyses; however, Ponda et al. [85] suggest that correcting for a deficiency might not translate into clinically meaningful changes in lipid concentrations. In addition, a systematic literature search revealed no statistically significant effects for vitamin D supplementation were observed for total cholesterol, HDL and triglycerides [86]. Therefore, the lipid modulating effects of vitamin D supplementation should be further investigated. Taken together, these findings suggest that vitamin D may improve lipid profiles in *ApoE* allele obese patients.

Vascular endothelial growth factor (VEGF) is abundantly secreted from adipocytes and plays a key role in the process of fat tissue formation through the regulation of angiogenesis. A positive correlation between the concentrations of circulating VEGF levels and BMI was demonstrated in healthy male subjects under highly controlled conditions [87]. Angiogenesis has been associated with visceral and subcutaneous adipose tissue in severe human obesity [88]. The treatment of mice with TNP-470, an angiogenesis inhibitor, reduced blood flow from the recipient into the graft after subcutaneous transplantation of epididymal fat. The weight of transplanted tissues and the size of adipocytes in the grafts were significantly lower in mice treated with TNP-470 (TNP mice) than in the control mice [89]. Different angiogenesis inhibitors have been shown to significantly decrease body and adipose tissue weights [90]. These studies demonstrate that adipose tissue mass can be regulated through the vasculature and that metabolic changes accompany anti-angiogenic-induced weight loss, which may contribute to weight reduction. VEGF expression in visceral fat is enhanced during growth and is related to fat deposition [91]. Serum VEGF concentrations have been positively correlated with BMI and visceral fat area [92]. In one experiment, a high fat-intake increased the VEGF mRNA expression in visceral fat and the VEGF concentration in plasma, accompanied with the increase in the plasma free fatty acids concentration in mice [93]. VEGF-B specifically controlled the endothelial uptake of fatty acids via transcriptional regulation of vascular fatty acid transport proteins [94]. Angiogenesis was inhibited by blocking the VEGF receptor 2 in mice with diet-induced obesity. These treated mice had significantly lower body weights than control animals [95]. The serum VEGF-A levels were significantly higher in obese patients than in lean controls, decreasing after weight loss with bariatric surgery [96]. *VEGF* haplotypes confer susceptibility to obesity in children and adolescents [97]. In contrast, calcitriol has been reported to inhibit angiogenesis *in vitro* and *in vivo*[98]. Calcitriol significantly inhibits VEGF-induced endothelial cell spouting and elongation in a dose-dependent manner and decreases the production of VEGF [99]. Calcitriol is a potent inhibitor of retinal neovascularization in a mouse model of oxygen-induced ischemic retinopathy [100]. Vitamin D and its analogs also reduce the expression of VEGF in various cancer cell lines [101,102]. Moreover, *DBP*-*maf* have been reported to inhibit angiogenesis and tumor growth in mice [103] and to inhibit VEGF signaling by decreasing VEGF-mediated phosphorylation of VEGF-2 and ERK1/2, a downstream target of the VEGF signaling cascade [104]. These findings suggest that vitamin D modulates angiogenesis in obesity.

Poly(ADP-ribose) polymerases (PARPs) comprise a family of enzymes sharing a conserved catalytic domain that support mono- or poly(ADP-ribosyl)transferase activity using NAD^+^ as a donor of ADP-ribosyl units. PARPs are involved in a wide range of molecular and cellular processes, including maintenance of genome stability, regulation of chromatin structure and transcription, cell proliferation, and apoptosis [105]. PARP-1 is a critical regulator of peroxisome proliferator-activated receptor gamma (PPARγ2)-dependent gene expression with implications in adipocyte function and obesity-related disease models. PARP-1 expression increases during adipocyte development [106]. *PARP*-*1*^−/−^ mice with predominantly obesity-resistant backgrounds were more susceptible to age-related weight gain and diet-induced obesity than wild-type littermates [107,108], while the decreased activity of PARP-1 in mice with obesity-prone background protected against high-fat diet-induced obesity [109-111]. These findings suggest that genetic determinants can modify the phenotypic outcomes of weight gain. *PARP*-*2*^−/−^ mice had defects in adipogenesis with reduced expression of adipogenic genes [112] and are also protected against diet-induced obesity [113]. *PARP*-*5* knockout mice also exhibited increased energy expenditure, increased fatty acid and glucose utilization, and reduced adiposity [114]. In addition, increased levels of vitamin D seem to down-regulate PARP-1 expression; PARP-1 levels decrease following calcitriol treatment in NB4 cells, which are acute promyelocytic leukemia cells [115]. Vitamin D exerts a concentration-dependent inhibitory effect on PARP-1 in human keratinocyte cells [116]. Vitamin D-induced down-regulation of PARP is further enhanced by nicotinamide in human myeloblastic leukemia cells [117]. Furthermore, PARP was attenuated in the hippocampi of rats that received dexamethasone and vitamin D [118], suggesting that the anti-inflammatory effects of dexamethasone and vitamin D are derived from their capacity to down-regulate microglial activation. These findings suggest that vitamin D may have a protective role in obesity by down-regulating PARP.

### The role of vitamin D in obesity

Matrix metalloproteinases (MMPs) are proteolytic enzymes that are responsible for remodeling the extracellular matrix and regulating leukocyte migration through the extracellular matrix. This migration is an important step in inflammatory and infectious pathophysiology. MMPs are produced by many cell types, including lymphocytes, granulocytes, astrocytes, and activated macrophages. There is growing evidence that MMPs play an important role in the pathogenesis of obesity. Bouloumié et al. [119] provided the first evidence that human adipose tissue releases MMP-2. Overweight/obese women had significantly higher plasma activity of MMP-2 than controls [120]. MMP-9 levels are found increased in obese subjects [121,122]. Significantly higher levels of MMP-9 have been reported in obese children with coexisting hypertension than in obese normotensive patients, and MMP-9 correlates with BMI [123]. In one study, obese children and adolescents had higher circulating MMP-8 concentrations, lower plasma tissue inhibitor of metalloproteinase 1 (TIMP-1) concentrations, and higher MMP-8/TIMP-1 ratios than non-obese controls [124]. An *MMP*-*2* promoter haplotype was associated with percentage of body fat in childhood obesity in New Zealand [125]. Functional *MMP*-*9* gene polymorphism is strongly associated with obesity [126], and *MMP*-*9* genotypes and haplotypes affect MMP-9 levels in obese children and adolescents [127]. Moreover, VDR-knock-out mice have been shown to have an influx of inflammatory cells, phospho-acetylation of NF-κB, and up-regulated expression of MMP-2, MMP-9, and MMP-12 in the lungs [128]. The *VDR TaqI* polymorphism is associated with the decreased production of TIMP-1, a natural MMP-9 inhibitor [129]. In addition, calcitriol modulated tissue MMP expression under experimental conditions [130], down-regulated MMP-9 levels in keratinocytes, and may attenuate the deleterious effects of excessive TNF-α-induced proteolytic activity, which is associated with cutaneous inflammation [131]. Calcitriol also inhibits both the basal levels and the staphylococcus-stimulated production of MMP-9 in human blood monocytes and alveolar macrophages [132]. Moreover, a vitamin D analog has also been reported to reduce the expression of MMP-2, MMP-9, VEGF, and PTH-related peptide in Lewis lung carcinoma cells [111]. Together, these studies suggest that calcitriol may play an important role in the pathological processes of obesity by down-regulating the level of MMPs and regulating the level of TIMPs.

The mitogen-activated protein kinase (MAPK) pathways provide a key link between the membrane-bound receptors that receive these cues and changes in gene expression patterns, including the extracellular signal-regulated kinase (ERK) cascade, the stress-activated protein kinases/c-jun N-terminal kinase (SAPK/JNK) cascade, and the p38 MAPK/RK/HOG cascade [133]. p38MAP kinase activity is required for human primary adipocyte differentiation [134]. Obese Zucker rats exhibit MAKP activity [54]. MAPK phosphatase-1 (MKP-1) plays an essential physiological role in the negative regulation of the MAPKs, and MKP-1 plays an important role in the regulation of energy expenditure and facilitates the loss of oxidative myofibers associated with obesity in mice [135]. Mice lacking MKP-1 expression are resistant to diet-induced obesity [136]. The down-regulation of MKP-1 is critical for increased production of MCP-1 during the course of adipocyte hypertrophy [137]. The exposure of obese mice to UV-B resulted in the phosphorylation of ERK1/2, JNK, and p38 proteins of the MAPK family. Compared to wild-type mice, obese mice exhibited higher levels of phosphorylation of these proteins, greater activation of NF-ĸB/p65, and higher levels of circulating proinflammatory cytokines [138], suggesting that obesity in mice is associated with greater susceptibility to UVB-induced oxidative stress. In both dietary and genetically (*ob*/*ob*) obese mice, adipose tissues displayed a marked decrease in p38MAPK activity compared with the same tissues from lean mice. Furthermore, p38MAPK activity is significantly higher in pre-adipocytes than in adipocytes [139], suggesting that p38MAPK activity decreases during adipocyte differentiation. The *MAP2K5*-linked single nucleotide polymorphism *rs2241423* was associated with BMI and obesity in two cohorts of Swedish and Greek children [140], suggesting a role for MAP2K5 in early weight regulation. By regulating VDR mRNA expression, the p38 MAPK pathway participates in the mediation of calcium signals and affects lipid accumulation in murine pre-adipocytes [141]. Pretreatment with calcitriol has been shown to inhibit JNK activation by all stressors and also to inhibit p38 activation in keratocytes [142]. Zhang et al. [143] demonstrated that the up-regulation of MKP-1 by vitamin D inhibited lipopolysaccharide (LPS)-induced p38 activation and cytokine production in monocytes and macrophages. In another study, the vitamin D analog (24R)-1,24-dihydroxycholecalciferol prevented neuronal damage caused by hydrogen peroxide-induced toxicity in the SH-SY5Y cell line [144]. Interestingly, the neurotoxic effects of hydrogen peroxide were dependent on JNK and p38 MAPK. In addition, the long-term actions of vitamin D in MCF-7 and LNCaP cancer cells can suppress the estradiol-induced activity of ERK-1 MAPK and inhibit cell growth [145].

The reduced form of the nicotinamide adenine dinucleotide phosphate (NADPH) oxidase (NOX) enzyme complex mediates critical physiological and pathological processes, including cell signaling, inflammation, and mitogenesis, by generating reactive oxygen species (ROS) from molecular oxygen. The up-regulations of p22^phox^ and p47^phox^ in adipose and Nox4, p22^phox^, and p47^phox^ in the kidneys has been observed in obese subjects [146]. Overweight and obese adults have increased vascular endothelial expression levels of NOX-p47^phox^ and evidence of endothelial oxidative stress, with selective compensatory upregulation of antioxidant enzymes and Ser1177-phosphorylated endothelial nitric oxide synthase [147]. The reduced expression of the NADPH oxidase NOX4 is a hallmark of adipocyte differentiation [148]. NOX4 acts as a switch between differentiation and proliferation in pre-adipocytes [149]. NOX4-deficient mice display latent adipose tissue accumulation and are susceptible to diet-induced obesity and early onset insulin resistance [150]. The knockdown of NOX4 by RNA interference inhibits reactive oxygen species production and adipocyte differentiation by differentiation-inducing agents [151]. The suppression of NOX2 may restore free fatty acids-induced dysfunction and apoptosis in β cells [152]. However, vitamin D deprivation in rats decreased the activity of cytosolic NADPH-dependent 3,5,3′-triodo-L-thyronine (T_3_) binding in the liver. This decrease can be restored by administering calcitriol [153]. In heart mitochondria, NAD^+^-dependent isocitrate dehydrogenase decreased notably in vitamin D-deficient rats, but calcitriol subsequently restored normal values [154]. In rat centrilobular hepatocytes, a vitamin D-deficient diet induced a significant increase in NADPH [155]. Husain et al. [156] reported that cardiac NOX activity increased by 300% in uremic rats compared with the normal controls. Treatment with paricalcitol protected the uremic rats from cardiac oxidative stress by inhibiting NOX activity (by 50%), thus lowering superoxide production in the heart. Taken together, these findings and results indicate that vitamin D may have a role in obesity via the suppression of NADPH expression.

Prostaglandins (PGs) play a role in inflammatory processes. Cyclooxygenase (COX) participates in the conversion of arachidonic acid into PGs. PGE_2_ and PGD_2_/PGJ_2_ have shown to promote adiposity in mice through the inhibition of lipolysis and the induction of adipogenesis, respectively [157,158]. PGF_2α_ is a potent inhibitor of adipocyte differentiation *in vitro*[159-161]. PGE_2_ enhanced lipid accumulation in hepatocytes and contributed to the development of hepatic steatosis in *in vivo* models of obesity [162]. Obesity-associated inflammatory foci in the human breast are associated with elevated COX-2 levels and activation of the PGE_2_[163]. In one study, a *COX*-*2* genetic deficiency produced in a significant reduction in total body weight and percentage of body fat. In adipose tissue, the production of the precursor required for 15d-PGJ_2_ formation, PGD_2_, was significantly reduced; macrophage-dependent inflammation was also significantly reduced [158]. Moreover, calcitriol has been reported to regulate the expression of several key genes involved in the PG pathway, causing a decrease in PG synthesis [164]. Calcitriol and its analogs have been shown to selectively inhibit the activity of COX-2 [165]. These findings suggest that vitamin D may play a role in modulating the inflammatory process in obesity.

Reactive oxygen species (ROS) are produced by activated phagocytes as a part of their microbicidal activities. An association between ROS and metabolic syndrome has been demonstrated in asymptomatic Japanese men [166]. Diet-induced obesity increases the levels of total and individual ROS in the brain and highlights a direct relationship between the amount of adiposity and the level of oxidative stress within the brain [167], suggesting that obesity increases cerebro-cortical ROS and impairs brain function. High-fat diets (HFDs) induce obesity and result in an increase in oxidative stress in adipose tissue. One study reported an increase in body weight after 90 days of HFD and observed that exercise training prevented greater gain. Lipid peroxidation and protein carbonylation increased in fat tissue after HFD and decreased significantly after exercise training [168], suggesting an interaction between ROS and lipolysis. Obesity has been linked to a low-grade pro-inflammatory state, in which impairments in the oxidative stress and antioxidant mechanisms are involved [169,170]. The over-expression of manganese superoxide dismutase (MnSOD) ameliorated high-fat diet-induced insulin resistance in rat skeletal muscle [171]. Obese adult rats experienced greater mitochondrial hydrogen peroxide release compared with lean adult rats [172]. *Glutathione S*-*transferase P1* gene polymorphisms increase the susceptibility to and risk of type 2 diabetes mellitus and obesity [173]. Significant impairment in glutathione peroxidase has been observed in response to diet-induced obesity [167]. The suppression of glutathione peroxidase activity was demonstrated in diabetic patients compared to healthy controls, and suppression was observed to occur in a greater degree in obese vs. non-obese diabetics. There were higher levels of oxidative stress in the obese diabetics even after control of hyperglycemia by insulin treatment, suggesting the importance of obesity in contributing to oxidative stress [174]. A significant decrease of all glutathione forms, including the content of total glutathione (GSH) and glutathionylated proteins, has been demonstrated in obese and type 1 diabetic children [175]. Similarly, calcitriol has been reported to exert a receptor-mediated effect on the secretion of H_2_O_2_ by human monocytes [176]. Human monocytes in culture gradually lose their capacity to produce superoxide when stimulated. The addition of calcitriol, lipopolysaccharide or lipoteichoic acid restored the capacity of stimulated monocytes to produce superoxide and increased their oxidative capacity compared with unstimulated monocytes [177]. Calcitriol can also protect nonmalignant prostate cells from oxidative stress-induced cell death by eliminating ROS-induced cellular injuries [178]. Vitamin D metabolites and vitamin D analogs have been reported to induce lipoxygenase mRNA expression, lipoxygenase activity and ROS in a human bone cell line [179]. In another study, the vitamin D analog (24R)-1,24-dihydroxycholecalciferol prevented neuronal damage caused by hydrogen peroxide-induced toxicity in the SH-SY5Y cell line [144]. Vitamin D can also reduce the extent of lipid peroxidation and can induce SOD activity in a hepatic anti-oxidant system in rats [180]. Astrocytes play a pivotal role in the CNS detoxification pathways in which GSH is involved in eliminating oxygen and nitrogen reactive species, such as NO. Calcitriol affects this pathway by enhancing intracellular GSH pools and significantly reduces the nitrite production that is induced by LPS [181]. These findings suggest that vitamin D modulates oxidative stress in obesity.

Nitric oxide synthase (NOS) is an enzyme that is involved in the synthesis of nitric oxide (NO), which regulates a variety of important physiological responses, including cell migration, the immune response, and apoptosis. High fat diets modulate nitric oxide biosynthesis and antioxidant defense in red blood cells in C57BL/6 mice [182], which lead to increasde NO sensitivity in rat coronary arterioles [183]. Platelet NO production has been significantly correlated with BMI, waist circumference, and triglyceride concentrations, thus suggesting an association between increased platelet NO production, obesity, and hypertriglyceridemia, independent of the degree of insulin-resistance [184]. Chronic NOS blockade by L-NAME in mice ameliorated high-fat diet-induced adiposity and glucose intolerance, accompanied by reduced adipose inflammation and improved insulin signaling in skeletal muscle, suggesting that endogenous NO plays a modulatory role in the development of obesity-related insulin resistance [185]. Mice deprived of the eNOS and/or nNOS gene exhibit metabolic syndrome, including insulin resistance, hypertension, and dyslipidemia [186]. Inducible NOS (iNOS)-null mice, although protected from obesity-related insulin resistance, exhibit increased adiposity [187]. Conversely, the activation of 1*α*-hydroxylase in macrophages increases the level of calcitriol, which inhibits the iNOS expression and reduces NO production within LPS-stimulated macrophages [188]. Thus, calcitriol production by macrophages may provide protection against the oxidative injuries that are caused by the NO burst. Calcitriol is known to inhibit LPS-induced immune activation in human endothelial cells [189]. In experimental allergic encephalomyelitis, calcitriol inhibits the expression of iNOS in the rat CNS [190].

## Conclusions

This paper reviewed the relationship between vitamin D and obesity. Genetic studies provide opportunities to determine which proteins link vitamin D to obesity pathology. Vitamin D is also able to act through numerous non-genomic mechanisms, including protein expression, oxidative stress, inflammation, and cellular metabolism. These findings suggest that vitamin D plays a role in obesity. However, some studies demonstrated no effect of vitamin D on weight change and energy expenditure and little improvement in cardiovascular risks with cholecalciferol [191-194]. Interestingly, Vitamin D is fat-soluble and readily stored in adipose tissue; it is sequestered after absorption and stored in tissues such as fat and muscle [195]. This fate of vitamin D has been demonstrated by injecting radio-labeled vitamin D_3_ into individuals and monitoring the highest levels of biological activity and radioactivity in the fat tissue [196]. In addition, cholecalciferol supplementation has no effect on cytokines and markers of inflammation in obese subjects [197]. Calcitriol, inversely, modulates adipokine expression, inhibits anti-inflammatory cytokine expression, reduces monocyte recruitment by human pre-adipocytes, and restores glucose uptake in adipocytes [198-200]. Calcitriol has a role in human adipose tissue because it is the active form of vitamin D_3_ metabolite, and VDR receptors present in adipocytes allow the suppression of PTH levels. The PTH excess observed in elderly subjects with both primary and secondary hyperparathyroidism may promote weight gain by impeding catecholamine-induced lipolysis [201]. However, monitoring of serum 25OHD after calcitriol intake is not necessary because calcitriol inhibits the production of serum 25OHD in the liver [202,203]. Taken together, there is widespread evidence from laboratory, animal and genetic studies that calcitriol is active in obesity. Calcitriol therapy in obesity has not been reported whilst the results of trials of cholecalciferol supplementation have so far been limited. Therefore, further investigation of calcitriol in obese patients is needed.

## Competing interests

The authors declare that they have no competing interests.

## Authors’ contributions

This work was carried out in collaboration between both authors. Author KL designed the study and wrote the protocol. Author LN managed the literature searches. All authors read and approved the final manuscript.
